# Impact of a Bio-Cross-Linking Agent Obtained from Spent Coffee Grounds on the Physicochemical and Thermal Properties of Gelatin/Κ-Carrageenan Hydrogels

**DOI:** 10.3390/ma17194724

**Published:** 2024-09-26

**Authors:** Paulina Sapuła, Paulina Zając, Krzysztof Pielichowski, Konstantinos N. Raftopoulos, Katarzyna Bialik-Wąs

**Affiliations:** 1 Faculty of Chemical Engineering and Technology, Department of Organic Chemistry and Technology, Cracow University of Technology, Warszawska 24, 31-155 Kraków, Poland; paulina.sapula@doktorant.pk.edu.pl; 2 Faculty of Chemical Engineering and Technology, Department of Chemistry and Technology of Polymers, Cracow University of Technology, Warszawska 24, 31-155 Kraków, Poland; paulina.zajac@pk.edu.pl (P.Z.); krzysztof.pielichowski@pk.edu.pl (K.P.)

**Keywords:** bio-cross-linking agent, coffee waste extract, hydrogels, gelatine

## Abstract

Gelatine hydrogels can be prepared using different cross-linking methods, such as enzymatic, physical or chemical. Unfortunately, in the case of chemical cross-linking, the typically utilized synthetic cross-linkers are harmful to human health and the environment. Therefore, in accordance with the principles of green chemistry and sustainable development, we have obtained compounds for the chemical cross-linking of hydrogel polymers from the processing of spent coffee grounds. In this study, gelatin/κ-carrageenan hydrogels are cross-linked using a bio-cross-linking agent from spent coffee grounds. Their physicochemical and thermal properties are compared with those of standard physical gels. The chemical cross-linking was confirmed based on FT-IR spectra, which demonstrated the formation of new covalent bonds between the oxidized polyphenols included in the extract from the spent coffee grounds and the amide groups present in the gelatine structure. Significant differences were also observed in morphology (SEM images) and other physico-chemical characteristics (gel fraction, swelling ability, hardness). The chemically cross-linked hydrogels in comparison to physically ones are characterized by a better developed porous network, a slightly higher gel fraction (64.03 ± 4.52% as compared to 68.15 ± 0.77%), and a lower swelling ratio (3820 ± 45% as compared to 1773 ± 35%), while TGA results show that they have better thermal stability. The research confirmed the possibility of using the developed natural cross-linking agent in the process of obtaining hydrogel materials based on bio-polymers.

## 1. Introduction

Hydrogels based on natural polymers are materials with a three-dimensional structure; one of the basic properties of these is the excellent ability to absorb fluids [1,2,3]. Due to their specific properties, such as elasticity, porosity, biocompatibility, and biodegradability, they are used in a wide range of fields, with particular emphasis on medicine and cosmetology [4,5,6].

The use of a combination of protein and polysaccharide polymers is an interesting approach because of their similarity to the extracellular matrix (ECM) in native tissues. Furthermore, these polymers can form secondary networks in non-covalent ways, such as hydrogen bonds, hydrophobic interactions, and electrostatic interactions [7,8,9,10]. The combination of these two types of natural polymers allows for, among other things, improvements in the thermal properties, mechanical strength, and stability of protein materials. The addition of polysaccharides is a method to modify the basic properties of proteins, and thus the final parameters of hydrogel materials, to adapt them to their potential applications [7,8,9,10].

Gelatin is a polymer obtained by the physical and chemical degradation or thermal denaturation of collagen [11]. It is characterized by biocompatibility, non-immunogenicity, low antigenicity, biodegradability, and a high capacity to absorb liquids. Its disadvantages include, among others, low thermal stability and insufficient mechanical properties [12,13]. Due to its properties, it is used in dressing materials, soft and hard capsules, drug delivery systems, and tissue engineering [13,14].

Meanwhile, κ-carrageenan is obtained by extracting red seaweed under alkaline conditions [15,16]. This polymer exhibits high hydrophilicity, mechanical strength, biocompatibility, biodegradability, and stimuli-responsive characteristics [17]. Due to specific properties such as nontoxicity, easy gelling, thermoreversibility, and significant viscoelasticity, polysaccharide-based hydrogels have found application in tissue engineering, drug delivery, and biosensors [17,18].

Chemical cross-linking is a method of obtaining hydrogels with increased stability and improved mechanical and thermal properties. In this process, a polymer or a mixture of polymers reacts with a low molecular weight cross-linking agent, resulting in the creation of new covalent bonds [19,20,21]. Among the commonly used gelatin cross-linking agents are glutaraldehyde, genipin, and 1-ethyl-3-(3-dimethyl aminopropyl)-carbodiimide [22,23,24]. The most commonly used synthetic compounds may be toxic to cells in the human body and, as a result, cause side effects that negatively affect health. This aspect excludes the use of these types of substances in materials intended for dermatological purposes, especially those in contact with broken or damaged skin [24]. Natural cross-linking agents obtained from plant raw materials are becoming an increasingly popular alternative [23,24].

Coffee waste is a raw material that is currently largely unutilized but has significant potential due to its commonness and abundance. Coffee waste may be present at various stages of the plant raw material processing, from the treatment of coffee berries through the roasting of coffee beans to the preparation of coffee drinks [25,26,27]. Spent coffee grounds are a source of many organic compounds, such as polysaccharides, oligosaccharides, lipids, aliphatic acids, amino acids, proteins, alkaloids and phenolics, minerals, lignin, melanoidins, and volatile compounds [25,26]. Phenolic compounds include chlorogenic, caffeic, ellagic, trans-ferulic, gallic, *p*-hydroxybenzoic, *p*-coumaric, protocatechuic and tannic acids, catechin, epicatechin, rutin, and quercetin [28,29].

Significant amounts of polyphenols present in the coffee waste material allow the use of its extract as a natural cross-linking agent for biopolymers to obtain hydrogel materials which, as in the case of gelatin, are considered dually cross-linked materials due to the presence of both physical and chemical interactions [11,12]. Polyphenols contain numerous functional groups in their structure, such as hydroxyl and carboxyl groups, which can react with amino groups of proteins and form covalent bonds [30]. The cross-linking of polymers occurs because of the oxidation of polyphenols to quinones, which then react with histidine and form covalent cross-linking through the Schiff base reaction and the Michael addition reaction [18,31]. Furthermore, polyphenols have a number of biological properties (antioxidant, antimicrobial, anti-inflammatory) that prove useful in pharmaceutical and cosmetic applications [30,31].

Hydrogels based on proteins and polysaccharides are cross-linked mainly using synthetic cross-linking agents, such as glutaraldehyde, carbodiimide, phosphate groups, boric acid, acrylamides, and epoxy compounds [18,23]. Many of these compounds may have toxic effects on human cells, which limits their use in the preparation of biomedical materials. Natural cross-linking agents such as genipin, enzymes, citric acid, tannic acid, phytic acid, and vanillin are becoming increasingly popular [18,23]. These compounds have much lower cytotoxicity compared to synthetic cross-linking agents and may also have additional properties beneficial for medical applications, such as antioxidant or anti-inflammatory effects [18,23]. The presented research refers to the above-described problem related to the need to develop materials for medical applications based on safe and non-toxic cross-linking agents. The conducted research aimed to determine the application potential of the developed cross-linking agent. The novelty of the described study was the use of a plant extract from waste raw material, namely spent coffee waste, as a cross-linking agent for hydrogel materials based on gelatin and κ-carrageenan.

Hence, our research was primarily aimed at investigating the possibilities of the application of coffee waste extract as a natural cross-linking agent and to determine its effect on the physicochemical and thermal properties of hydrogel materials based on gelatin and κ-carrageenan. A basic characterization of physical properties was carried out, concerning the determination of the degree of cross-linking, the swelling ratio, and the hardness of the hydrogels. Moreover, to confirm the occurrence of the chemical cross-linking reaction of the polymers, FT-IR analysis was performed, and the influence of the bio-cross-linking agent on the morphological structure of the materials was determined on the basis of SEM micrographs. TGA and DSC tests were a source of information on the molecular mobility and thermal stability of the hydrogels and allowed for the comparison of physically cross-linked materials with samples containing the addition of a natural cross-linking agent.

## 2. Materials and Methods

Gelatin from porcine skin (Bloom 300, Type A, Solubility 50 mg/mL, CAS: 9000-70-8) was supplied by Sigma-Aldrich (St. Louis, MO, USA) and κ-carrageenan (Solubility 50 mg/mL, CAS: 11114-20-8) was obtained from Pol-Aura (Olsztyn, Poland). Buffer solution (pH 10 ± 0.05, Composition: boric acid, disodium tetraborate, potassium chloride, Relative density: 1.02 g/cm^3^ for 20 °C) was purchased from Chempur (Piekary Śląskie, Poland). Spent coffee ground samples generated after espresso coffee preparation from light-roasted coffee (100% Arabica), were obtained from local sources. The material was air-dried and stored at room temperature in hermetic containers prior to analysis.

**Figure 1:**
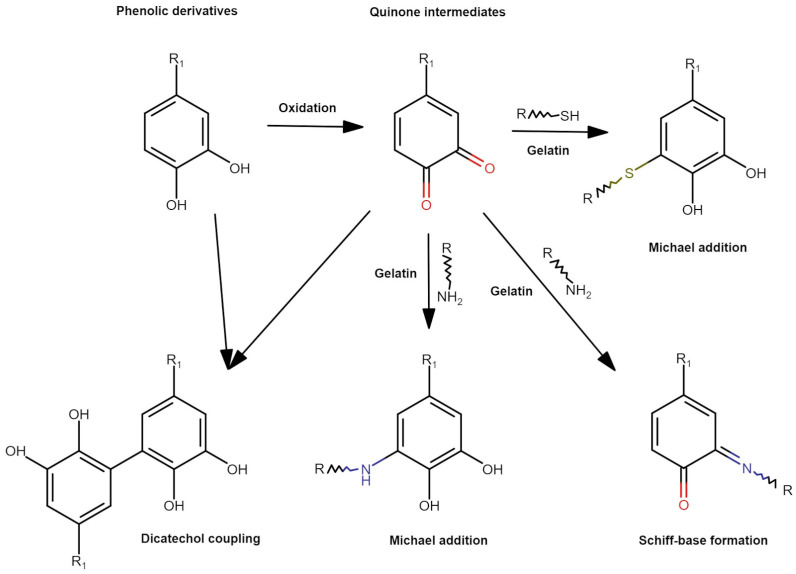
Postulated schematic presentation of gelatin cross-linking reactions using phenolic compounds.

Hydrogel materials were obtained based on a mixture of two natural polymers—gelatin (protein) and κ-carrageenan (polysaccharide). The applied cross-linking agent was a plant extract obtained from spent coffee grounds using an ultrasound/microwave-assisted extraction method. This is a modern extraction method combining both ultrasound and microwaves. The combination of two effective extraction methods allows for the additional reduction of the duration and increase in the efficiency of the process. The use of ultrasound is responsible for increasing the depth of solvent penetration into the plant sample and for increasing contact surface area. Microwaves allow for quick heating of the extraction mixture, thus increasing the mass flow and solubility of active compounds in the solvent [32]. The potential routes in the gelatin cross-linking process are shown in Figure 1.

In order to perform the extraction process, 15% (*w/v*) of spent coffee grounds with a particle diameter in the range of 0.25–0.5 mm, a buffer solution with pH 10, and rapeseed oil in a volume ratio of 19:1 (*v/v*) were used. The extraction process was conducted in three stages: (1) ultrasound-assisted extraction; (2) microwave-assisted extraction; (3) ultrasound-assisted extraction. During the first and third stages, the extraction mixture was exposed to ultrasound with a power of 120 W and a frequency of 40 kHz for 10 min at a temperature of 40 °C. However, the second stage was based on the use of microwave radiation with a power of 140 W, a temperature of 60 °C, and atmospheric pressure. The obtained extract was filtered through a filter cloth and then centrifuged in a laboratory centrifuge at a speed of 4000 rpm. Finally, compressed oxygen was bubbled through the spent coffee grounds extract for 30 min at room temperature [33]. The described procedure was developed on the basis of preliminary research conducted to select a method and favorable parameters for the coffee waste extraction process. The abbreviation “extract” is used later in the publication for spent coffee grounds extract.

The cross-linking process was performed by adding the extract prepared in accordance with the abovementioned procedure to the selected polymer/polymer mixture with a pH 10. The concentration of gelatin used in the final mixture was 1.75% (*w/v*), and the concentration of κ-carrageenan was 0.5% (*w/v*). The mixture was stirred for 10 min and then exposed to compressed oxygen for 30 min at a temperature of 60 °C. The mixtures were poured into Petri dishes and allowed to fully cross-link and dry in ambient conditions. The reference material consisted of samples obtained using the method described above, but without the addition of a cross-linking agent [34].

**Figure 2:**
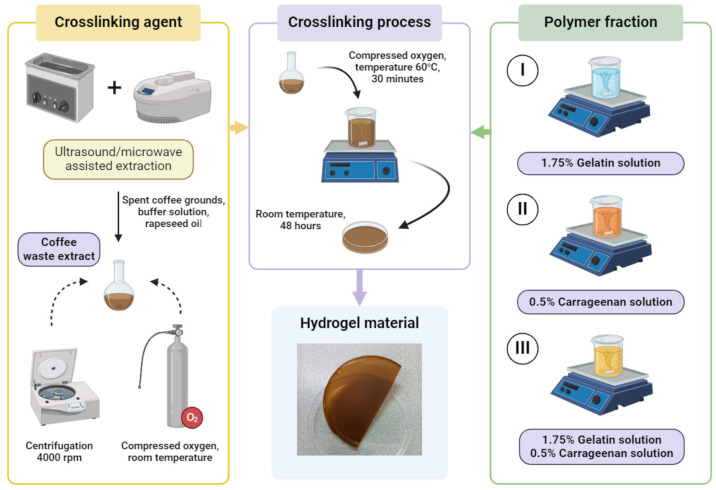
The process of obtaining hydrogel materials cross-linked with coffee waste extract.

A diagram of the process for obtaining hydrogel materials cross-linked with coffee waste extract is shown in Figure 2.

Table 1 presents a list of the obtained hydrogels, which were subjected to further tests.

To determine the gel fraction, three samples with dimensions of approx. 10 × 10 mm were cut from all obtained hydrogels and conditioned at 40 °C for 24 h and weighed (*W*_0_). After that, they were immersed in distilled water at room temperature for 48 h up to an equilibrium swelling weight. Then, the hydrogel samples were conditioned again at 40 °C for 24 h and weighed once more (*W_e_*). The gel fraction (%*GF*) was calculated according to the following Equation (1):

(1) $$\%GF=W_{e}/W_{0}\cdot 100\%$$

where:

*W*_0_—the weight of the dry sample before the test (g),

*W_e_*—the weight of the dry sample after the test (g).

The swelling ratio (%*SR*) was evaluated for all hydrogel samples with the mass of approximately 0.250 g, which were immersed in excess distilled water at room temperature. The dry weight (*W_d_*) of the samples was determined prior to the test. The swollen samples were removed and weighed (*W_s_*) after 1 and 24 h, after previously removing the surface water using filter paper. For each of the obtained hydrogels, three measurements were averaged. The swelling ratio (%) of all the tested hydrogel samples was determined using the following Equation (2):

(2) $$\%SR=\left(W_{s}-W_{d}\right)/W_{d}\cdot 100\%$$

where:

*W_s_*—the weight of the dry sample before swelling (g),

*W_d_*—the weight of the sample after swelling (g).

The hydrogel hardness was tested according to the PN-ISO 868 standard [35] using a type A Shore durometer (Insize Co., Loganville, GA, USA). The measurements were carried out at ambient temperature and data were recorded 15 s after the pressing probe touched the specimen. Each sample was subjected to quintuple testing on both sides of the material and the data are shown as a mean ± standard deviation.

To investigate the chemical structure of the obtained hydrogel materials, attenuated total reflection (ATR) Fourier transform infrared (FT-IR) spectroscopy was conducted. The measurements were performed with a Nicolet iS5 Thermo Scientific spectrophotometer (Waltham, MA, USA) with an ATR attachment equipped with a diamond crystal. The absorbance spectra were acquired over a range of 400–4000 cm^−1^ at ambient temperature.

The morphology of hydrogel materials was investigated by scanning electron microscopy (SEM) using an Apreo 2 S LoVac instrument (Thermo Fisher Scientific, Waltham, MA, USA) equipped with a Schottky electron emission source operating at an acceleration voltage of 2.00 kV. For the high vacuum mode, the microscope was equipped with an Everhart-Thornley Detector (ETD) operating in BSE mode. The sample was sputtered with 2.5 nm gold and attached to the stage with carbon tape.

The thermal stability of the materials was studied under an inert atmosphere (nitrogen) using a Netzsch TG 209 F1 Libra thermogravimetric analyzer (Selb, Germany). Samples of the environmentally equilibrated materials of 3–5 mg were measured in alumina crucibles (Al_2_O_3_). The scanned temperature range was 30–600 °C and the heating rate 10 K/min.

DSC curves were recorded with a Mettler Toledo 823 e differential scanning calorimeter purged with argon (Greifensee, Switzerland). Samples of 8 to 10 mg equilibrated under ambient conditions, as described in the synthesis section above, were placed in standard aluminum crucibles and were subjected to the following protocol with heating/cooling rates 10 K/min. A first run to 100 °C was performed to remove any residual water. Then, the samples were cooled down to −45 °C and finally they were heated up to 120 °C. As no features, except for water evaporation, are visible in the first heating and cooling cycle, here only the second heating will be shown. The reported glass transition temperatures ($T_{g}$) are midpoint values.

## 3. Results and Discussion

### 3.1. Gel Fraction

**Figure 3:**
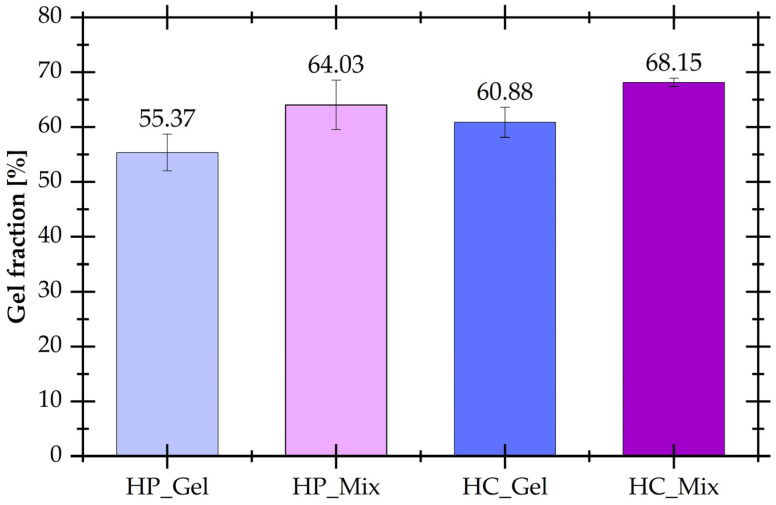
Gel fractions of hydrogels physically and chemically cross-linked.

The determination of the gel fraction allowed for comparison of the degree of cross-linking of hydrogels obtained physically (HP series of materials) and chemically (HC series of materials) using coffee extract as a cross-linker. Research results for all hydrogels are presented in Figure 3.

In gelatin hydrogels, various types of interactions occur, such as physical, chemical, or most often both [36]. In the case of physical gels, collagen-type triple helices are created when the solution is cooled. The gel fraction values show that hydrogels physically cross-linked are characterized by lower cross-linking degrees, such as 55.37 ± 3.33% and 64.03 ± 4.52% for HP_Gel and HP_Mix, respectively. Chemically cross-linked gels were prepared using the additional reagent, i.e., the natural bio-cross-linker made from coffee waste extract. It can be noticed that after the introduction of this cross-linking agent, HC_Gel and HC_Mix hydrogels have a higher share of the gel fraction, such as 60.88 ± 2.73% and 68.15 ± 0.77%. The increase in the value of the gel fraction is caused by a cross-linking reaction, which occurs in hydrogel formulations between the oxidized polyphenols included in the extract and the amide groups present in the gelatin structure. The presence of these new chemical covalent bonds was also confirmed by FT-IR spectra analysis. It turned out that for hydrogels based only on kappa-carrageenan, samples were completely soluble in distilled water and gel fractions were not determined, which can result from their lower concentration in the formulation before the reaction. Probably, kappa-carrageenan creates only interpenetrating networks with gelatin.

### 3.2. Determination of Swelling Ratios

**Figure 4:**
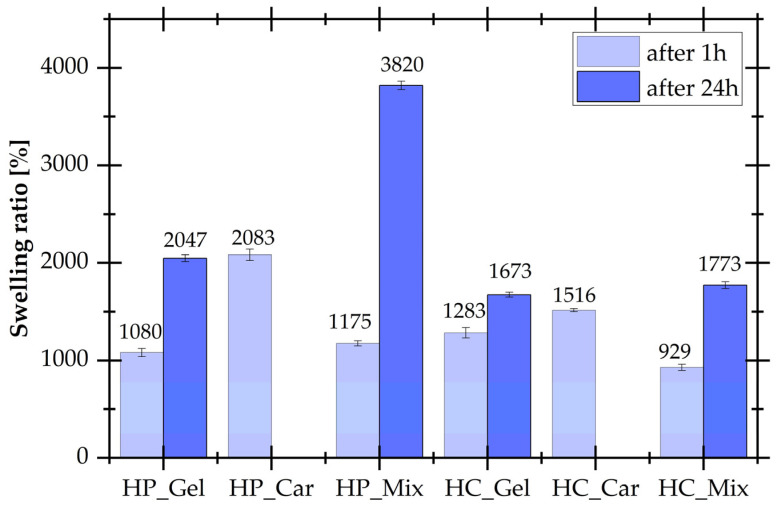
The dependence of swelling ratio on time for hydrogels physically and chemically cross-linked.

The swelling ability values for all hydrogel samples in distilled water are shown in Figure 4.

Generally, the swelling abilities in distilled water are better for hydrogels physically cross-linked. The highest swelling ratio [%] (3820 ± 45%) was determined for HP_Mix after 24 h, while for the same composition chemically cross-linked by coffee waste extract, this parameter decreases significantly (1773 ± 35%). It results from the chemical structure, cross-linking degree, and interactions between the polymer and the solvent. The higher the gel fraction of hydrogels, the lower the swelling capacity [37]. In the case of hydrogels physically cross-linked, only weak hydrogen interactions and interpenetrating networks appear. However, in the presence of coffee waste extract, chemically cross-linked hydrogels were prepared, which contain additional covalent bonds, and the spaces between the cross-linked sites become more and more occupied with polymer chains, therefore significantly reducing their degree of swelling. It was also observed that for hydrogels based only on κ-carrageenan, samples were completely soluble in distilled water after 24 h, but this is caused by the uncross-linked structure of the gel.

### 3.3. Hardness

**Figure 5:**
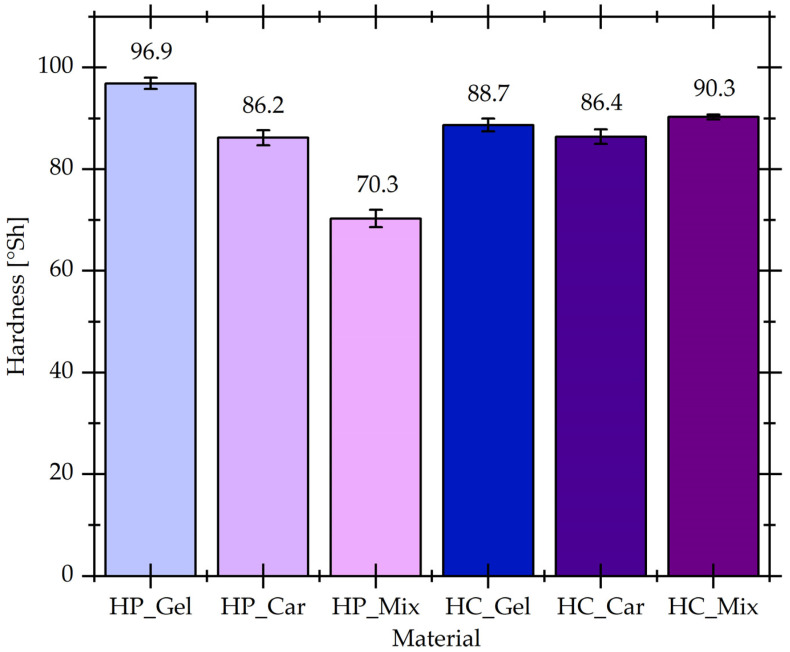
Hardness of physically and chemically cross-linked hydrogel materials.

The degree of cross-linking of the material may have a significant impact on the value of the hardness parameter, and the obtained results constitute an additional source of information on the effectiveness of polymer cross-linking reactions. Figure 5 presents the results of the hardness analysis of hydrogel materials cross-linked physically and chemically using coffee waste extract.

Based on the analysis of the gelatin-based hydrogel material cross-linked physically (HP_Gel) and chemically (HC_Gel), a slight decrease in the parameter value was observed for the sample containing the addition of a natural cross-linking agent from 96.9 ± 1.1 to 88.7 ± 1.3. In the case of κ-carrageenan hydrogels, the hardness value remained constant, 86.1 ± 1.5 and 86.4 ± 1.4 for HP_Car and HC_Car, respectively. No significant effect of the cross-linking agent on the hardness of the gelatin and carrageenan hydrogel materials was observed. Gelatin is a macromolecule with an extensive network of intermolecular interactions among protein structures involving, inter alia, hydrogen-bonding and hydrophobic interaction, which affect the mechanical properties of the material formed in the sol-gel transition [38,39]. As a result of the formation of a three-dimensional structure stabilized by various types of physical interactions, the HP_Gel sample showed a hardness value comparable to the HC_Gel hydrogel containing the bio-cross-linking agent; however, this was not compatible with the higher degree of cross-linking of the HP_Gel material. The values for the HP_Car and HC_Car samples reflect the lack of cross-linking between κ-carrageenan and the cross-linking agent, which is also confirmed by the results of the degree of cross-linking and the degree of swelling. In addition, a significant increase in the parameter from 70.3 ± 1.7 to 90.3 ± 0.5 was observed for the gelatin–carrageenan mixture cross-linked with coffee waste extract (HC_Mix). Gelatin interacts with κ-carrageenan via the interaction of charged gelatin and polysaccharide macroions, which leads to the formation of (bio)polyelectrolyte complexes [40]. The introduction of a bio-cross-linking agent into the polymer mixture causes a chemical cross-linking reaction to occur and the HC_Mix material with improved physicochemical properties is obtained. The study allowed us to observe a significant decrease in the hardness of the HP_Mix sample in relation to the gelatin material caused by the use of κ-carrageenan additive, which has a plasticizing effect and increases the elasticity of the hydrogel.

### 3.4. Attenuated Total Reflectance Fourier Transform Infrared Spectroscopy (ATR-FTIR)

**Figure 6:**
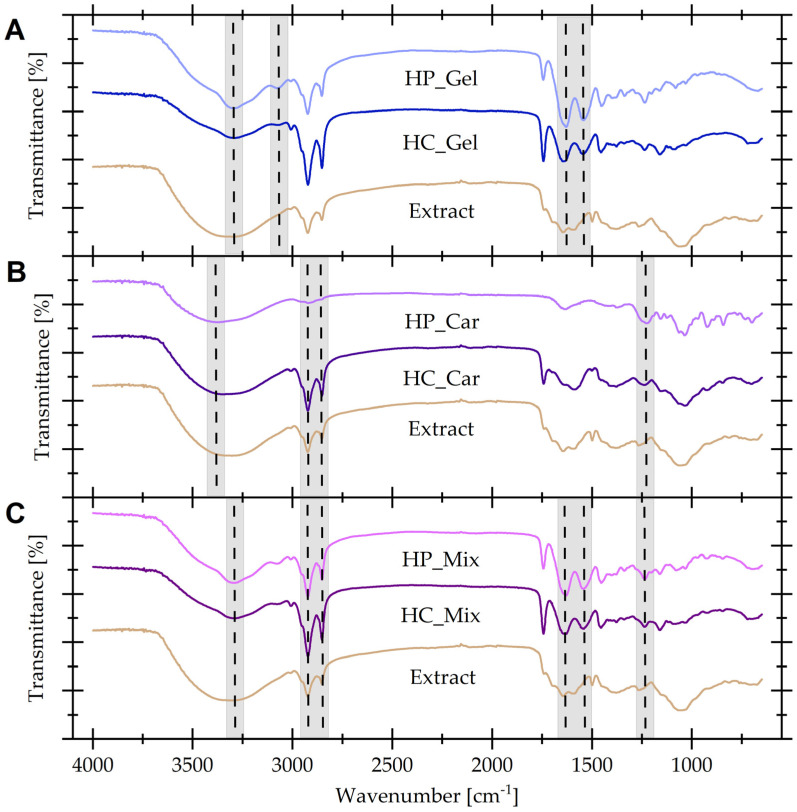
FT-IR spectra of physically and chemically cross-linked hydrogel materials containing: (**A**) gelatin; (**B**) κ-carrageenan; (**C**) gelatin–carrageenan. Dashed lines and gray backgrounds indicate peaks discussed in the text, which show significant changes in the chemical structure of hydrogels.

Figure 6 shows the FT-IR spectra of the samples with and without the addition of the spent coffee grounds extract, which acts as a natural cross-linking agent.

The spectrum of gelatin material without the addition of extract showed absorption bands corresponding to structures characteristic of the pristine gelatin, such as amide A (3291 cm^−1^), amide B (3078 cm^−1^), amide I (1629 cm^−1^), amide II (1543 cm^−1^), and amide III (1235 cm^−1^). Furthermore, there were bands originating from asymmetric vibrations of the C–H bond in the CH_2_ group (2922 cm^−1^) and symmetric vibrations of the C–H bond in the CH_3_ group (2853 cm^−1^). The addition of a natural cross-linking agent resulted in some changes visible in the spectrum. There was a significant reduction in the transmittance of the band at the wavenumber of 3290 cm^−1^ from 54% to 86%, the band at the wavenumber of 1643 cm^−1^ from 34% to 62%, and the band at the wavenumber of 1543 cm^−1^ from 42% to 70%. Additionally, there was a disappearance of the band corresponding to the amide B structure and a significant shift of the band characteristic of the amide I structure to a wavenumber of 1643 cm^−1^ with a simultaneous decrease in signal intensity. The observed changes may indicate the occurrence of a chemical cross-linking reaction of gelatin through the formation of amide bonds between the oxidized polyphenols present in the extract and the amide groups present in the protein structure. Moreover, the decrease in the transmittance of the band at a wavenumber of 3290 cm^−1^ indicated slighter signal coming from O–H stretching vibrations, which can be identified with the additional participation of these bonds in the cross-linking process [11,41,42].

The IR spectrum of the extract-free κ-carrageenan hydrogel contains a number of characteristic bands corresponding to O–H stretching vibrations (3368 cm^−1^), C–H stretching vibrations of CH_2_ (2921 cm^−1^), structural water deformation (1633 cm^−1^), S = O bond of sulfate esters (1225 cm^−1^), bridge O stretching vibrations (1159 cm^−1^), glycosidic linkage (1035 cm^−1^), C–O bond of 3,6-anhydrogalactose (923 cm^−1^) and C–O–SO_3_ bond on D-galactose-4-sulfate (842 cm^−1^) [17,41,43,44,45,46,47]. As a result of the use of the cross-linking agent, the transmittance of the band at the wavenumber 3348 cm^−1^ and 2923 cm^−1^ increased significantly, and additionally, at the wavenumber of 2853 cm^−1^, a new band appeared, corresponding to the symmetric vibrations of the C–H bond in the CH_3_ group. Moreover, the absorption band at the wavenumber 1237 cm^−1^ was shifted while the signal intensity increased. The described changes can be explained directly based on the spectrum of the cross-linking agent, where the following bands appear: O–H stretching vibrations (3303 cm^−1^), C–H stretching vibrations of CH_2_ (2923 cm^−1^), C–H stretching vibrations of CH_3_ (2853 cm^−1^) and O–H deformation vibrations of carboxyl groups or N–H bending vibrations (1265 cm^−1^) [17,41,43,44,45,46,47].

The spectrum of the gelatin–carrageenan material without the addition of a cross-linking agent includes absorption bands originating from both the structure of gelatin and κ-carrageenan. It showed bands typical of amide A (3291 cm^−1^), C–H stretching vibrations of CH_2_ (2923 cm^−1^), C–H stretching vibrations of CH_3_ (2853 cm^−1^), amide I (1629 cm^−1^), amide II (1542 cm^−1^), amide III (1235 cm^−1^), bridge O stretching vibrations (1160 cm^−1^) and C–O bond of 3,6-anhydrogalactose (925 cm^−1^). As a result of the cross-linking reaction, a slight increase in band transmittance was observed at wavenumbers of 2922 cm^−1^ and 2852 cm^−1^ caused by the presence of an additional signal from the cross-linking agent. Moreover, there was a visible decrease in the transmittance of the absorption bands at 1629 cm^−1^, 1542 cm^−1^, and 1235 cm^−1^ resulting from the possible occurrence of a chemical cross-linking reaction, as in the case of gelatin material, between the oxidized polyphenols included in the extract and the amide groups present in gelatin. The decrease in transmittance at a wavenumber of 3291 cm^−1^ from 61% to 82% may additionally indicate the potential participation of OH groups in the cross-linking process of the hydrogel material [41,48].

### 3.5. SEM Analysis

**Figure 7:**
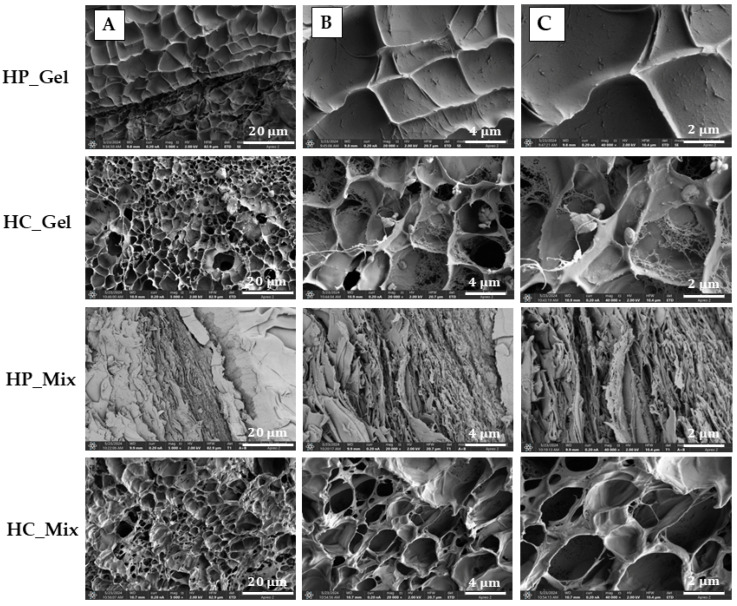
SEM images of the cross-section of hydrogels physically and chemically cross-linked using different magnifications: (**A**) 5000×, (**B**) 20,000×, (**C**) 40,000×.

In order to visualize the microstructure of the cross-section of hydrogels physically and chemically cross-linked, scanning electron microscopy (SEM) analysis was conducted (Figure 7).

Comparing the structure of chemically and physically cross-linked hydrogels (Figure 7), it can be seen that they are very different. The physically cross-linked hydrogel, such as HP_Mix, has a layered, quite regular structure. The cross-sectional view of this hydrogel sample clearly shows that it does not have any open pores. However, chemically cross-linked hydrogels (HC_Gel and HC_Mix) are loose and large networks with ordered nano- and micropores embedded in them. In addition, it can be noticed that they are characterized by a sponge-like structure, with high porosity and an open network. Some internal networks can be observed in the pores, which probably indicates an additional chemical cross-linking of the gelatin, especially in the case of HC-Gel. Moreover, the structure in the case of HC_Mix is less regular, and pores are more varied in size from 0.5 up to even 4 µm. Carvalho and Mansur [49] observed that the reduction in pore size was attributed to polymerization reactions leading to covalent cross-linking and a slowing down in chain mobility. Interestingly, the average pore size of the obtained chemically cross-linked hydrogels is significantly smaller than in the case of hydrogels obtained by photopolymerization [49] or by using microbial transglutaminase [50]. From the point of view of drug delivery systems, this could be an interesting effect, because such structures and morphologies of chemically cross-linked hydrogels probably will enable the controlled and prolonged release of drugs.

### 3.6. Thermogravimetric Analysis (TGA)

**Figure 8:**
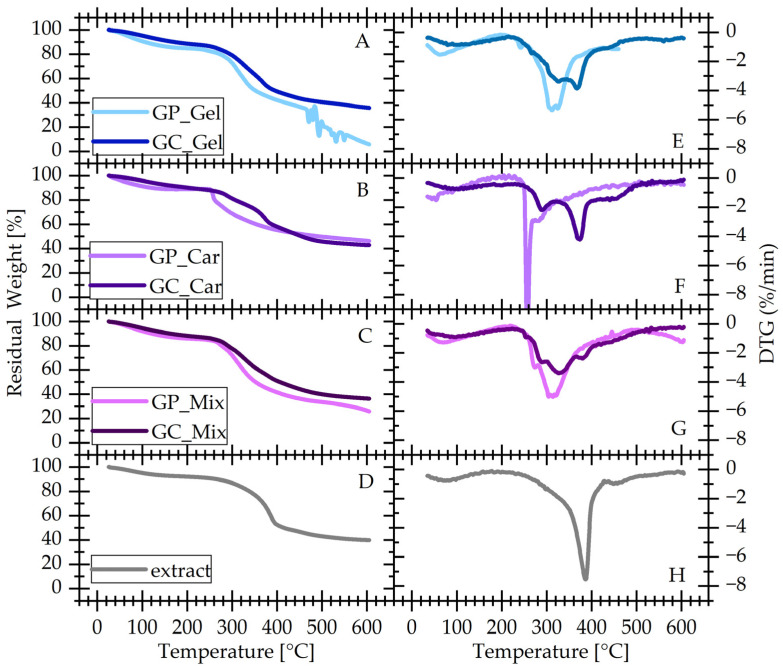
(**A**–**D**) Thermogravimetric (TGA) and (**E**–**H**) differential thermogravimetric curves of all materials under investigation grouped by natural polymer as described in the legends. Panels D and H show the curves recorded with the extract.

Figure 8A–D shows the thermogravimetric curves for all gels under investigation, grouped by the type of the used natural polymer, along with the curve for the extract.

Starting from low temperatures, the first step, extending approximately up to 150 °C, is associated with the release of moisture by the gels and the extract. For all gels, the absorbed moisture is in the range of 15 wt%, and the extract appears also hydrophilic with an absorption of ~8 wt% under ambient conditions. It is interesting to note that this step, along with the corresponding DTG peaks (Figure 8E–G), migrates to higher temperatures with chemical cross-linking progress, indicating tighter binding of water in the cross-linked systems.

The materials are thermally stable up to 240 °C (Figure 8). The extract (Figure 8D–H) shows a major, sharp degradation step at 385 °C and a weaker one at 453 °C, as quantified by the peak temperatures of the DTG signal (Table 2). These steps persist, as expected, in the cross-linked gels, without significant changes in their peak temperature.

The polymeric components degrade at lower temperatures, with gelatine being more stable by approximately 40 degrees in a non-blended and non-cross-linked state. The peaks associated with the degradation persist in the mixed and cross-linked systems as standalone peaks. Cross-linking improves the thermal stability of the base polymers, presumably because it hinders the mobility of the system and thus the reactions leading to degradation. This phenomenon is more pronounced for κ-carrageenan. The mixing of gelatine and κ-carageenan does not have any marked effect on the thermal stability of either component.

### 3.7. Differential Scanning Calorimetry (DSC)

**Figure 9:**
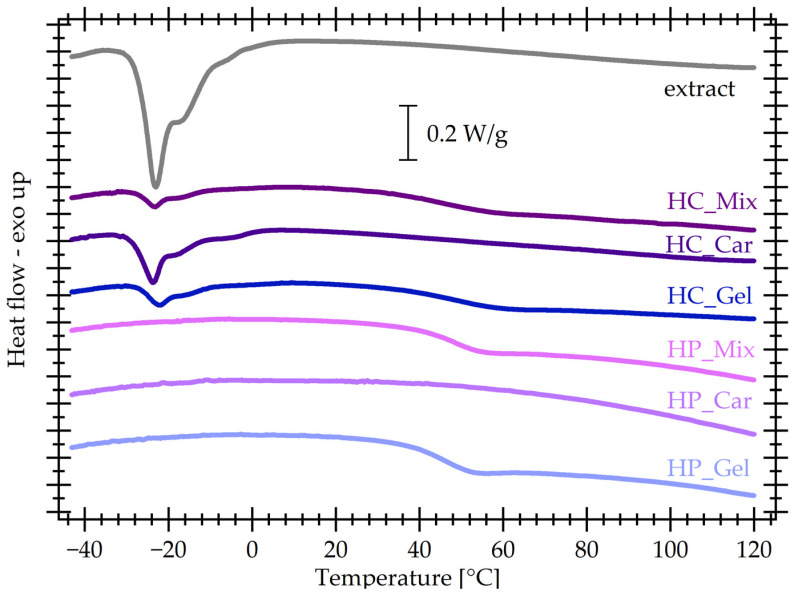
DSC curves recorded with all gels under investigation, in dry form. The curve of the extract itself is included for comparison.

Figure 9 shows the second heating DSC curves for all materials under investigation. For the gelatine-containing gels, a step associated with the glass transition is visible between 40 and 50 °C. The gels based on κ-carrageenan, both physically and chemically cross-linked, lack any transitions in the studied temperature range. However, the introduction of κ-carrageenan in the composition of the physical gel increases, by a few degrees, the glass transition temperature (Table 2), indicating a moderate slowing of molecular mobility, which is attributed to the bulkier nature of its structural units. Chemical cross-linking of gelatine causes an increase in glass transition temperature as compared to the physical gel, along with a broadening of the step, and a decrease in the heat capacity change Δ*c_P_*, which is consistent with a slowing down of mobility with cross-linking [51]. However, the step of the cross-linked mixed systems at a temperature similar to that of the non-cross-linked gelatine is significantly broader, indicating a quite high dynamic heterogeneity consistent with the cross-linking.

Interestingly, at temperatures around −20 °C, chemically cross-linked systems show a complex endothermic peak. The phenomenon associated with this is not clear at this point, but it appears also in the curve of the extract, and hence it should be related with substances present in it.

The conducted studies confirmed the possibility of utilizing spent coffee grounds extract as a bio-cross-linking agent in the process of obtaining hydrogel materials based on natural polymers, such as gelatin and κ-carrageenan. In further stages of the research, the composition of the reaction mixture and the parameters of the cross-linking reaction will be optimized, and studies on the release of active substances and in vitro tests will be conducted.

## 4. Conclusions

A series of hydrogels based on gelatin, κ-carrageenan, and a gelatin–carrageenan mixture cross-linked with phenols present in the extract of coffee waste, and their properties, were compared to equivalent systems cross-linked solely by physical interactions. The introduction of a bio-cross-linking agent into the polymer fraction was successful in obtaining chemically cross-linked materials, as confirmed by FT-IR and DSC results. The highest degree of cross-linking and, at the same time, the lowest degree of swelling was found for the HC_Mix material, which was a gelatin–carrageenan mixture cross-linked by the bio-derived cross-linking agent. Moreover, a positive effect of the cross-linking agent content on both of these parameters was observed both in the case of gelatin and its mixture with kappa-carrageenan. In the case of both κ-carageenan-based materials (HP_Car and HC_Car), a complete dissolution occurred, indicating that there was no cross-linking reaction with the coffee waste extract. Despite this, the addition of κ-carrageenan in the mixture may have some technological interest due to its plasticizing effects observed by the hardness test. In addition, the material consisting of a mixture of polymers underwent the chemical cross-linking process most effectively. The FT-IR analysis confirmed the occurrence of a certain degree of chemical cross-linking of gelatin and the gelatin–carrageenan mixture. This was determined based on the shifts and changes in the transmittance of absorption bands characteristic of the gelatin structure such as amide A, amide I, amide II, and amide III. SEM micrographs also confirmed the significant influence of the cross-linking agent on the cross-sectional morphology of hydrogel materials. A particular change was observed for the HC_Mix, which, as a result of the presence of coffee waste extract, changed its structure from compact and layered to a less regular but highly porous form. Cross-linking had a positive effect on the thermal stability of the polymers, which could be related to the changes observed in morphology. The gelatin–carrageenan hydrogel cross-linked with a natural cross-linking agent from coffee waste exhibits significant application potential as a modern dressing material. Therefore, in the next stages of research, it is planned to conduct studies on the release of active substances and in vitro tests.

## Figures and Tables

**Table 1:** Composition of hydrogel materials cross-linked with a natural cross-linking agent.

Sample	Gelatin [% *v/v*]	Κ-Carrageenan [% *v/v*]	Cross-Linking Agent [% *v*/*v*]
HP_Gel	100	-	-
HP_Car	-	100	-
HP_Mix	67	33	-
HC_Gel	67	-	33
HC_Car	-	50	50
HC_Mix	50	25	25

HP—physically cross-linked hydrogel, HC—chemically cross-linked hydrogel.

**Table 2:** DTG peak temperatures and glass transition characteristics for all materials under investigation.

Sample	*T_max_* (°C)		*T_g_* (°C)	Δ*c_P_* (J/gK)
	Polymer	Extract		
HP_Gel	317	-	44.0	0.68
HP_Car	278	-	-	-
HP_Mix	275, 314	-	46.7	0.61
HC_Gel	320	366, 450	47.4	0.42
HC_Car	288	371, 452	-	-
HC_Mix	288, 329	378, 454	43.7	0.47
extract	-	385, 453	-	-

## Data Availability

The original contributions presented in the study are included in the article, further inquiries can be directed to the corresponding authors.
